# The Dislike of Hot Thermal Conditions and Its Relationship with Sun (Ultraviolet Radiation) Exposure in the Southeastern United States

**DOI:** 10.3390/ijerph15102161

**Published:** 2018-10-01

**Authors:** Alan E. Stewart, Michael G. Kimlin

**Affiliations:** 1 College of Education, University of Georgia, Athens, GA 30677, USA; mkimlin@usc.edu.au; 2 Health Research Institute, University of the Sunshine Coast, Brisbane 4558, Australia; 3 Cancer Council Queensland, Brisbane 4006, Australia

**Keywords:** adults, attitudes, hot temperature, melanoma, risk-taking, skin neoplasms, sunbathing, sunlight, temperature, ultraviolet rays

## Abstract

We investigated the relationship between peoples’ preferences for being outside during certain months of the year, based upon their dislike of hot or warm temperatures, and of taking precautions against ultraviolet radiation (UVR) exposure. A sample of university undergraduates (*N* = 1400) living in the Northern Hemisphere completed an online survey in the late summer of 2017 that inventoried their dislike of heat and hot conditions, their sun tanning preferences and habits, and their preferences for being outside during different months of the year, along with whether they would protect themselves from the UVR exposure during those months. Dislike of hot conditions was negatively correlated with respondent preferences for sun tanning and with the number of months during the year that people enjoyed being active outside. A greater proportion of people who disliked hot conditions experienced risks of UVR overexposure during the spring and fall. In contrast, people who expressed more liking of heat frequently enjoyed being outside during the warmer months (April to October), and a significantly greater proportion of them experienced risks for sun overexposure in these months. Such individual differences in heat-related attitudes may explain a proportion the variability in individual risk behaviors for skin cancer that is not currently accounted for by approaches using objective variables such as temperature, thermal comfort indices, or the UV index.

## 1. Introduction

In 2014 the US Surgeon General issued a call to action for the prevention of skin cancer in the American population. Specifically, the focus of the call to action was the reduction of exposure to ultraviolet radiation (UVR), as this is the most preventable risk factor for developing skin cancer [1]. At the time of the Surgeon General’s call, 76,665 people in the United States were diagnosed with melanoma and 9234 people died as a result of this cancer. In the USA, melanoma ranks as the fifth most prevalent cancer among men and sixth among women [2]. The incidence of melanoma in non-Hispanic whites has increased significantly from 2005 to 2014. Although incidence rates have increased for people from ages 15 to 44 years, the rates also have increased for people aged 55 years and older [3]. Globally, the incidence of melanoma in 2015 was 351,880, with an age-standardized rate of five cases occurring per 100,000 persons [4]. Older men that reside in the Australasian, North American, and European regions experience the greatest burden for melanoma [4].

Melanoma is caused by severe intermittent sun exposure, sunburn, or use of tanning beds [5,6]. Occupational sun exposure and total lifetime sun exposure also are associated with melanoma risk in people living at low latitudes [7]. Although some sun exposure may be accidental or inadvertent, some people seek sun exposure to obtain a suntan. Additionally, some people use UV tanning beds to obtain a suntan, and this can increase melanoma risks. Young women (aged 14–30 years) typically have been at the most risk for melanoma from tanning beds [8]. Laws in the United States and Canada have increasingly required either parental consent or the meeting of minimal age requirements to use a tanning bed [9,10]. Although this has led to a decrease in tanning bed use among young women, people still seek a tan through sun exposure [9]. 

Intermittent sun exposure and severe sun exposure (or overexposure) leading to sunburn typically occur during the warmer summer months when solar radiation is the most intense, and when warmer ambient air temperatures permit outdoor recreational activities, often as people expose greater areas of skin (e.g., hiking, swimming, tennis, etc.) [11]. Some occupations also have intermittent sun exposure, such as teachers on playground duty [12]. Some lower latitude locations are so warm during the summer that this may contribute to a decrease in the amount of time that most people spend outside, and the sun protection measures that they use [13]. 

Anecdotally, the authors have observed that people vary in the seasons that they experience risks for sunburn according to their subjective heat preferences, and how this relates to their being outside. Here, thermal perceptions refer to the individual differences that exist in the interpretation and preference for a given thermal environment. Such individual differences in heat perception and preference are part of behavioral thermoregulation, where people try to balance comfort, health, and the performance of work or recreational tasks by their behavioral choices of environments [14]. 

Beyond these observations, two studies have suggested that people who dislike heat and hot conditions may experience heightened risks for sunburn the during spring and fall months when milder ambient air temperatures afford relief from summertime heating during recreational activities [8,10]. In this regard, objectively-based measures such as temperature, heat balance models, or the recently derived Universal Thermal Comfort Index (UTCI) may not fully explain a person’s likelihood of being outside and getting a sunburn [15,16,17]. In addition to seasonal considerations, the individual variability in sun exposure as it relates to temperature and thermal comfort suggests that individuals may differ in the extent which they dislike or prefer being in warm or hot outdoor environments [17,18,19,20]. The implication for sun exposure and skin cancer prevention is that those who like heat and hot conditions may experience greater risks for sunburn during the summer months. Further, people who dislike heat and hot conditions may experience greater sunburn risks during the spring and fall, but less during the summer. This reasoning is supported by research that found that peoples’ perceptions of solar radiation (sun sensation) contributed the most to their thermal sensations in outdoor spaces [21].

Individual differences in heat perception and preferences for thermal environments exist for several reasons. First, people may differ in the thermal environments to which they are acclimatized [14,16,17,20]. People from subtropical and tropical locations are acclimatized to higher temperatures compared to people from mid- and high-latitude regions. Importantly, culture and environmental attitudes have been observed to affect thermal perceptions of comfortable temperatures [20]. Second, the existing biometeorological indices of thermal comfort have been developed around average values for variables such as body surface area, body weight, percentage of body fat, skin wittedness, and cardiac output, among other things [22]. The use of average anthropometric values in models of thermal comfort suggests that the individual variability in perceived heat may not be accounted for. In addition, models of heat stress have been challenged in predicting individual observed sweating rates, and thus the rate at which a person may regulate cooling [22]. Third, and relatedly, an essential component in models of human thermal comfort is the individual metabolic rate [14]. Averages of metabolism are frequently used in thermal models, yet the metabolic rate can be quite variable and can depend upon one’s level of physical fitness and thyroid functioning, among other things [14,23,24]. 

Finally, existing thermal comfort models have not included higher-order psychological processes such as significant past experiences of temperature extremes, attitudes, beliefs, and the contributions of mood and emotion [17,20,25]. Some people may not feel or experience the degree of heat and the associated discomfort that has been normatively associated with a given biometeorological index. Further, it is possible that if they experience the heat and their body’s response to it (e. g., intense perspiration), they are not bothered by this or and do not negatively evaluate the environment as too warm or too hot. Alternatively, other people prefer cooler or milder temperatures for outside activities and also may believe (erroneously) that this temperature regime is associated with fewer risks for sunburn [15]. In this article, we report the results of a study of the relationships of subjective heat perceptions and preferences with the use of sun exposure precautions. We adopted the perspective of Knez and colleagues that personal and psychological variables may exert an important role along with weather, place, and time (here, months of the year) in affecting peoples’ sun protection behaviors [25]. 

Our experiences and this review of the literature suggested three hypotheses to us, the first of which was that people would differ individually in their subjective preferences for warm/hot environments. Some people will exhibit an affinity for heat while others will not. Second, we hypothesized that people with a higher level of dislike for heat and hot conditions would pursue sun exposure experiences less frequently or to a lesser extent than people who prefer warm conditions. Third, we expected that peoples’ perceptions and preferences for heat would affect when during the year that they may be more susceptible to sun over-exposure. Specifically, people who dislike heat and hot conditions may be at risk for sun over exposure during the early spring and later fall months when ambient air temperatures are less warm. Conversely, people who express an affinity for warm or hot outside conditions may experience greater risks for over exposure during the entire warm/hot season of summer.

## 2. Materials and Methods 

### 2.1. Participants and Procedures

The participants were 1400 undergraduate and graduate students (970 women, 430 men) recruited from a large public university in southeastern United States (Athens, Georgia, Latitude 33.93 °N, Longitude 83.32 °W). Historically, approximately 80% the students attending this university come from Georgia, with another 7% of students coming from within the southeastern United States (Arkansas, Alabama, Florida, Mississippi, North or South Carolina, Tennessee or Texas). The research project was reviewed and approved by the University of Georgia’s Institutional Review Board (approval: STUDY00005042). The participants completed the survey measures online after they were contacted with an initial message inviting their involvement. All people gave their informed consent to participate in the research. The incentive for participating in the research was a chance to receive one of five $50 Amazon gift cards. 

### 2.2. Measures

#### 2.2.1. Heat Perceptions and Preferences

We created an online survey with 15 items that assessed a person’s self-reported dislike of hot outside conditions. The first author’s prior work on the salience of weather environments informed the development of the thermal preference survey items [26]. Some items focused upon self-perceptions of heat: (a) I do not like the way hot air temperatures make me feel. (b) I do not like the sensation of feeling hot. Other items emphasized the respondent’s preferences to avoid heat: (a) being outside in warm weather makes me feel uncomfortably hot. (b) I avoid being in the sun during warm weather because the sunlight makes me feel too hot. The study participants used a five-point fully-anchored rating scale (1 = Strongly Disagree to 5 = Strongly Agree) to respond to these heat-related items. The survey measure and the instructions for how to score it appear in the Supplementary Materials for this article. As the items of the survey show, we have defined warm or hot conditions ipsatively (i.e., self-referentially) rather than objectively. Our reasoning for this definitional approach is that at least some aspects of behavior, especially as this concerns sun exposure, will come from the individual person’s subjective perceptions of conditions and how they feel in those conditions. 

The items displayed good internal consistency (Cronbach’s α = 0.96). In addition, we conducted a factor analysis of the 15 items, treating the respondents’ ratings as ordinal-level data (i.e., responses as ordered categories). The factor analysis yielded a single factor that related to the preferences for avoiding warm/hot outside conditions because people sensed and perceived that they were too warm/hot. Because the items all contributed to the assessment of this construct, we summed item ratings to create composite scores that possessed a possible range 15 to 75, with higher scores indicating a greater dislike of heat. 

#### 2.2.2. Outside Preferences, the Non-Use of Sunburn Protective Measures and Tanning Habits

In addition to heat dislike, we asked the respondents to indicate which months of the year that they enjoyed being outside because of the general weather conditions associated with that month. We also asked them to indicate the months during which they would take no protective measures to prevent sunburn when outside. Such measures included, for example, seeking shade, using sunscreen wearing protective clothing, wearing sun glasses, and/or wearing a hat. Next, we asked the respondents about their attempts to obtain a suntan during the previous (immediately past) summer season and to their preferences for tanning (1 = Do not like getting a suntan at all to 5 = Very much like getting a suntan). Related to this, we also asked participants to indicate their typical preferences for amount of tan (i.e., dark tan to practically no tan). Finally, we inquired about the participants’ uses of tanning salons (ever and within the last 12 months). 

#### 2.2.3. Demographic Variables 

The final portion of the survey collected respondent demographic information. We asked the participants to supply their age, to indicate their biological gender, and to indicate their primary racial and ethnic identifications. 

#### 2.2.4. Climate and UVR of the Research Location

Because this research examined the respondents’ perceptions and preferences for heat and hot thermal conditions as these were related to sun exposure, and because approximately 87% of the respondents come from Georgia or other locations in the southeastern United States, it is important to describe the climate of the research location at Athens, Georgia. This location and much of the southeastern United States experience subtropical humid conditions according to the Köppen–Geiger climatic classification [27]. Overall, the climate of Athens is temperate with no defined dry season, because the conditions are uniformly moist year-round. Summers are hot and muggy while winters are mild [27]. Table 1 summarizes the monthly temperatures, relative humidity, sunshine percentages, and sky cover [28]. Although the spring (March, April, and May) and fall (September, October, and November) seasons include pleasant and mild conditions, the summers at this location tend to be sunny and hot, especially considering the average levels of relative humidity. 

Table 2 shows the UV index (UVI) climatology for this region. The nearest measurement site is located 115 km from Athens, at Atlanta Ga. This small difference in distance does not significantly impact the UVI. Based on the World Health Organization’s (WHO) guidelines, this region experienced from high to extreme UVI conditions for 63.5% of the days of the year, which indicates this site is a high UVI location [29,30]. Importantly, even in the early spring (i.e., March and April) and in the fall (i.e., September and October), a majority of the days include UVI values that are at a high level or above. The WHO guidelines recommend that people use sun protective measures any time the UVI is at a level of 3 or higher. 

### 2.3. Data Analysis

We calculated descriptive statistics for all of the study variables. To examine the first hypothesis that the study participants would vary in their level preferences of warm or hot environments, we also constructed frequency histograms and assessed the extent to which the distribution of heat dislike scores approximated the standard normal distribution. We also checked for differences in heat dislike according to gender and race. We employed chi-square tests of independence and between-subject analyses of variance to examine these potential score differences. In examining the second hypothesis that differences in heat dislike would relate to differences in sun exposure and tanning behavior, we employed between-subject analyses of variance. Because our analyses of variances were used to examine differences in heat preferences and perceptions in existing groups (i.e., according to race, tanning preferences and histories), the groups often had unequal numbers of participants. For this reason, we interpreted the Type III sums of squares in evaluating the analysis of variance results. We used Spearman rank order correlation coefficients to assess relationships of preferences for sun tanning and the number of months spent outside with no sun protection with heat preferences and perceptions. Our third hypothesis involved examining how a dislike of heat affected the ways that people exposed themselves to the sun during each month of the year. We classified people as disliking heat (*N* = 369) if their scores on the 15 heat preference items were greater than or equal to the third quartile (i.e., score of 63). Similarly we classified people as heating liking (*N* = 352) if their scores were less than or equal to the first quartile (37) on the heat dislike items. We examined the proportion of each group of people (heat disliking and heat liking) who indicated that they enjoyed being outside because of the typical weather conditions during each month. We also noted the proportion of people in each group who took precautions (asked as a yes/no question) to prevent sunburn during each month. We constructed 95% confidence intervals for these proportions as a way to assess the extent to which the two groups (heat disliking versus heat liking) differed with respect to their outside preference and sunburn protection behaviors across the 12 months of the year. 

## 3. Results

### 3.1. Sample Characteristics

A summary of the sample characteristics appears in Table 3. The research participants ranged in age from 17 to 64 years, *M* = 24.0 years, *SD* = 6.38 years. An analysis of variance to examine age differences by gender and race was statistically significant, *F* (9, 1388) = 2.97, *p* = 0.0016. Although the main effect for gender was non-significant, we observed that the mean age for those identifying as Asian American (*M* = 22.7 years, *SD* = 3.6) were significantly younger than Caucasian American (*M* = 24.4, *SD* = 6.6, *p* = 0.018) and people with race identified as Other (*M* = 25.3, *SD* = 6.5, *p* = 0.004). A chi-square analysis of race and gender did not reveal any differences in the proportion of men and women within each category of race, *Χ*^2^ (*N* = 1398, *df* = 4) = 3.30, *p* = 0.51.

We observed differences in the participants’ preferences for obtaining a suntan in general, (1 = Do not like getting a suntan at all to 5 = Very much like getting a suntan). A main effect existed for gender, *F* (1, 1388) = 15.56, *p* < 0.0001. Women (*M* = 2.72, *SD* = 1.39) indicated a greater preference for obtaining a tan than did men (*M* = 2.22, *SD* = 1.18). A main effect for race also existed, *F* (4, 1388) = 25.34, *p* < 0.0001. African Americans (*M* = 1.71, *SD* = 1.22) exhibited less liking/preference for sun tanning than other groups (all *p* < 0.01). In addition, Caucasian Americans (*M* = 3.03, *SD* = 1.28) exhibited the highest preferences for tanning. These preferences were significantly greater than those for Asian Americans (*M* = 2.64, *SD* = 1.41), and for those identifying racially as Other (*M* = 2.19, *SD* = 1.27).

### 3.2. Individual Differences in Heat Perceptions and Preferences

**Figure 1:**
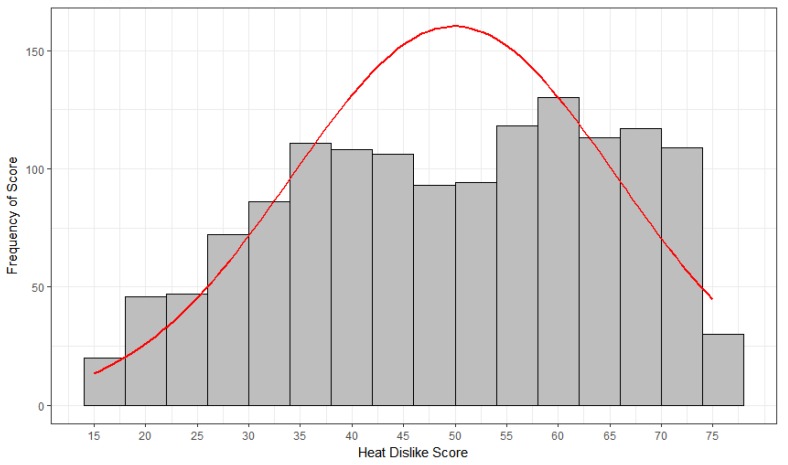
Frequency distribution of the total score on the Heat Dislike survey items with superimposed normal curve. (Note: Higher scores indicate greater dislike of heat).

People exhibited individual differences in their total scores of heat perceptions and preferences; Figure 1 shows the frequency distribution of the scores. The distribution exhibited slight negative skew (*Sk* = −0.21) and negative kurtosis (*Ku* = −1.01, i.e., a platykurtic distribution). With respect to measures of location, *M* = 49.9, *Mdn* = 51, and *Md* = 40. Scores in the sample ranged from 15 to 75, *SD* = 15.7. The distribution of scores appeared bimodal and exhibited statistically significant departures from the standard normal curve according to the Shapiro–Wilk test (*W* = 0.98, *p* < 0.0001); Figure 1 also shows a superimposed normal curve. Thus the distribution of scores cannot be considered normal.

Although men and women did not differ in their heat perceptions and preferences, we observed statistically significant differences according to race, *F* (4, 1397) = 4.20, *p* = 0.002, *η*^2^ = 0.02. Because two people did not report their race information, the total degrees of freedom were 1397. Persons who reported their race as Other (*M* = 55.1, *SD* = 14.1) expressed a significantly greater dislike of heat and hot conditions than did Caucasian Americans (*M* = 48.7, *SD* = 15.6, *p* = 0.0001).

### 3.3. Relationship of Heat Preferences and Sun Exposure Behaviors

The respondents’ dislike of heat and hot conditions were correlated negatively with both their preferences for getting a suntan (*r_s_* = −0.35, *p* < 0.0001) and with the total number of months of the year that they enjoyed being outside because of the weather conditions (*r_s_* = −0.39, *p* < 0.0001). The respondents were asked to indicate how they would expose themselves to the sun if they had the opportunity to spend time outside during the summer in a tropical place. There were four nominal choices, which ranged from avoiding the sun completely to sunbathing several hours each day (see Table 3). We assessed the extent to which these preferences for sun exposure may relate to the respondents’ heat preferences, by conducting a between-subjects analysis of variance. We treated the sun exposure preferences as a categorical independent variable, and we used the respondents’ dislike of heat and hot conditions as the dependent variable. People exhibited significant differences in heat dislike according to how they would expose themselves to the sun, *F* (3, 1396) = 47.46, *p* < 0.0001, *η*^2^ = 0.09. Table 4 shows the descriptive statistics for heat dislike according to category of sun exposure. People who would avoid the sun at all times evidenced the greatest dislike of heat compared to people who would sunbathe for several hours each day. 

There were 685 respondents who indicated that they had attempted to obtain a suntan during the preceding summer season of 2017; the remainder (*N =* 715) did not (see Table 3). People who attempted to obtain a suntan (*M* = 45.7, *SD* = 14.8) expressed significantly less dislike of heat and hot conditions than did those who had not sought a suntan (*M* = 53.9, *SD* = 15.5), *F* (1, 1399) = 101.32, *p* < 0.0001, *η*^2^ = 0.07. 

### 3.4. Relationship of Heat Preferences to Seasonal Outside Preferences and Use of Sunburn Protection

**Figure 2:**
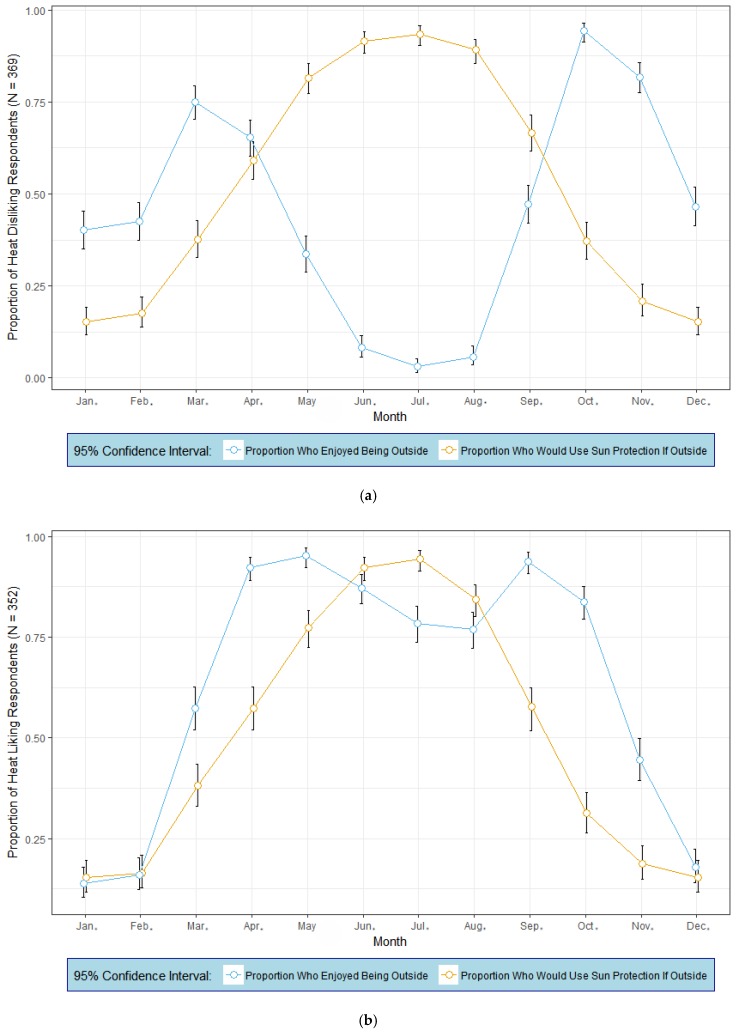
Mean proportions and 95% confidence intervals of respondents who enjoyed being outside (blue profile) and the mean proportion of respondents who would use sun protection if outside (orange profile, by month). (**a**) Heat disliking respondents (*N* = 369). (**b**) Heat liking respondents (*N* = 352). Note: Sun protection includes behaviors such as seeking shade, wearing sunscreen, wearing protecting clothing, sunglasses, and/or a hat.

We examined how the dislike of heat affected the ways that people exposed themselves to the sun during each month of the year. The results for those who disliked heat (*N* = 369) and those who did not express dislike of the heat (*N* = 352) appear in Figure 2a,b, respectively. Within each figure we plotted the mean proportions and the 95% confidence intervals of people who enjoyed being outside during each month and also plotted the mean proportion of people who reported that they would use protection *if they were outside* (e.g., sun screen, seeking shade, wear protective clothing, hats, etc.). People who disliked heat and hot conditions exhibited bimodal preferences for the months of the year that they enjoyed being outside: March (75%) and October (94%, Figure 2a). This group was most at risk for sunburn during the early spring (March and April) and mid fall (October), because the proportions of people taking sunburn precautions was significantly less (as illustrated by the 95% confidence intervals around the mean proportions in the figure) than the proportion who reported the enjoyment of being outside in those months. For this location, the UV index on a clear day in March rises above a value of 3 (moderate risk of harm for unprotected skin) at 11 am, and falls below 3 by 4 pm [31]. Similarly, the UV index is at or above 3 from 10 am to 4pm in April, and follows a similar pattern in October. It is possible that heat-disliking people may experience risks into the fall and early winter at this location if they remain outdoors for extended periods and do not take precautions; hence increasing the possible risks of overexposure. In contrast, people who disliked heat experienced comparatively lower sunburn risks in the May–September interval because of the small proportion of people who enjoyed being outside during the summer (e.g., only 4% in July). Nearly all of the people in this group reported that they would use sun protection during the mid-summer *if they were outside* (although very few people in this group enjoy being outside at this time of the year).

Figure 2b shows that the group of respondents who liked heat exhibited a substantially different monthly profile of preferences for being outside during the warmer months of the year. Preferences for being outside peaked in April (92%) and May (95%), ebbed somewhat during June, July, and August, and then peaked again in September (93%). July and August tend to be the hottest and the most humid months of the year at the research location (see Table 1); consequently even among people who like warm conditions, a smaller proportion of them may enjoy being outside in the hottest part of the summer. Importantly, in both spring (March, April, and May) and fall (September and October), the proportion of people preferring to be outside far exceeded, statistically, the proportion of them who reported that they would take precautions to prevent sunburn if outside during those same months. More so than the respondents who disliked heat, people who liked heat showed a susceptibility to overexpose themselves in the spring and fall. 

**Figure 3:**
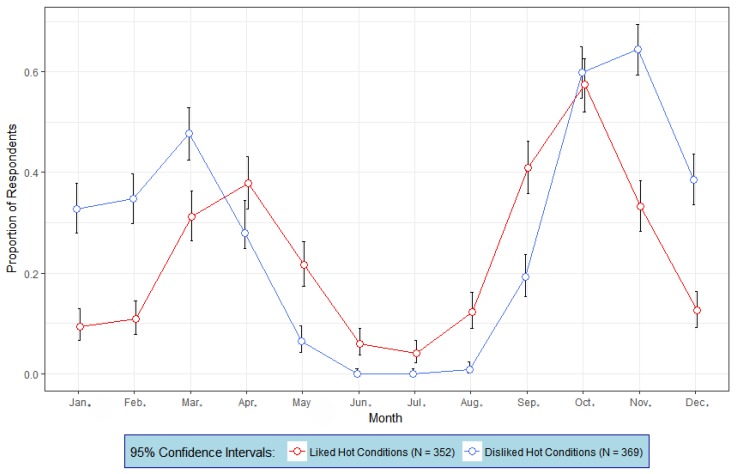
Mean proportions of heat liking respondents (*N* = 352) and heat disliking (*N* = 369) respondents who enjoyed being outside *and would use no sun protection if outside* (by month). A 95% confidence interval is also shown around each mean proportion.

How do people who dislike heat compare with those who like heat with respect to sun-exposure risk-taking? Figure 3 depicts the mean proportions of people from each group (dislike heat and like heat) who enjoy being outside each month and would not take any precautions to prevent sunburn. Consistent with the results in Figure 2a,b, those who disliked heat experienced the greatest risks for over-exposure in the early spring (March and April), and again in the fall (October and November). Those who liked heat experienced the greatest risks for over-exposure from mid-spring (April) to mid-fall (October). From April to September, a dip in both the profiles shows that regardless of heat liking or disliking, there are comparatively smaller proportions of people in each group that are outside and take no sunburn precautions. Nonetheless, a significantly greater proportion of people who liked heat experienced risks for over-exposure compared to those who disliked heat. Further, the risks for over-exposure by heat disliking participants were essentially nonexistent in June, July and August (i.e., the northern hemisphere summer season). It is possible, however, that heat disliking people may experience some risks for over-exposure in the morning before conditions become hot. The reason for this is that UV index at the research location rises above a value of 3 by 9 a.m. in June and July, and by 10 a.m. in August [31].

## 4. Discussion

We found that people possessed individual perceptions about both their thermal environments and how their bodies respond to such conditions. These perceptions also give rise to preferences (liking or disliking) for heat or hot outside environments. As such, the items of our survey constitute a measure of attitudes about hot outside conditions [20,25,32]. Moreover, the 15 items in the survey exhibited a high internal consistency and suggested that, taken together, they provide a useful indication for the extent to which people disliked heat or hot conditions. To our knowledge this represents the first study of its kind to assess heat attitudes, especially a dislike for hot outside conditions, and to relate them to UVR exposure and to taking precautions against sun (UVR) over-exposure and hence skin cancer prevention.

We also observed that people differed individually in their disliking of heat. Although the distribution of scores deviated from that of a standard normal distribution, the sample of participants exhibited a wide range of heat-related preferences, with most exhibiting a moderate degree of dislike of hot conditions. We attempted to understand the source of the score non-normality by examining the score distributions separately by gender and race. This did not produce any significant improvements in the degree of fit with a normal distribution. One possible explanation for the score distribution that we observed stems from the fact the research site was located within a subtropical humid climatic region [27]. Sampling a region that has a more balanced climatic year with winters that are as cold as the summers are hot may yield a more normal distribution of scores with respect to dislike of hot conditions. 

Perhaps most importantly, the results of this project showed that there were statistically significant relationships of dislike of heat and hot conditions with both past tanning behavior and in the times of year that people preferred to be outside. The dislike of heat and hot conditions shared from 7% of the variability with suntanning in the previous year to 14% of the variability in peoples’ attitudes towards obtaining a suntan. These results were noteworthy because they related to choices people made, or may make, about UVR exposure, simply because they perceived ambient thermal conditions to be aversive. For people with a higher dislike of heat, the risks for UVR overexposure are minimized at the outset because they tend to avoid or minimize sun exposure. At the opposing end of the continuum, once people choose to suntan, issues regarding the use of sunscreen, hats, and protective clothing become much more salient in avoiding sunburn. 

Suntanning behavior pertains to deliberate efforts to darken the skin due to sun exposure. However, what about more general exposure to UVR when outdoors during different months and seasons? We discovered that people had very different monthly UVR exposure profiles according to their individual dislike of heat. In this study, the respondents who disliked heat experienced more risks for overexposure in April, May, and October—more people were outside in these months than those who used sun protective measures, potentially due to the local climatic conditions at that time. The group of respondents who did not express a dislike of hot conditions evidenced a much wider interval of months in which they enjoyed being outside—from April through to October. It was only in the summer seasons (June, July, and August) that a higher proportion of people in this group tended to use sun protection than those who enjoyed being outside.

The authors are pursuing two lines of further research based upon the results that we reported here. First, we are interested in understanding what might contribute to individual differences in subjective thermal perception. We have designed another study in which we will examine the contributions of: 1. the climate in which one developed as a child and adolescent, 2. the person’s physical activity levels and physical fitness level, 3. body mass, 4. basal metabolic rates, and 5. levels of thyroid hormone. These variables may together explain some of the individual differences we observed here in thermal perceptions [14,23,24]. Second, we want to explore the sunburn risk perceptions and how these may be related to a like or dislike of warm or hot thermal environments [20,25].

This study was limited in that it relied upon participant self-reports to assess all of the variables. Additionally, the study design was cross-sectional. Another limitation pertains to the demography of the sample, who resided in a lower latitude climate of the southeastern United States. The survey responses of people who reside in cooler, higher latitude regions with a long winter and a short summer may well differ from those of the participants in the present sample. Finally, because we were interested in peoples’ subjective perceptions of thermal environments, we did not assess at this exploratory phase the contributions of physical variables such as body mass or activity levels. Our work is limited in this way; however, we will examine those contributions in a subsequent study. 

Despite these limitations, the study was unique both in assessing attitudes on hot thermal conditions and sun exposure, and in documenting that individual differences existed. Our emphasis on individual attitudes and sun exposure preferences may help to explain a small but unique portion of the variability in individuals’ risk behaviors for melanoma that are not currently accounted for by approaches that use objective variables such as temperature, heat balance models, thermal comfort indices, or UV indices [17,18,20,25]. In this regard, our approach is responsive to the recent call for novel and interdisciplinary approaches to understand and reduce melanoma risks [33].

## 5. Conclusions

This research focused on the assessment of the dislike of heat and its contributions to exposing oneself to the sun through the year. We observed that thermal preferences were related to taking precautions with the sun. Namely, those who liked being outside in hot conditions used sun protection proportionately less than people who disliked heat (Figure 3). This is a significant issue that warrants further investigation. The World Health Organization recommends that sun protection be used when the UV index is 3 or higher. The average winter UV index for this study site is 3. Perhaps this difference relates to a desire to tan, greater interests in participating in summer-season recreational activities outdoors, or wearing more revealing clothing in the summer season [34]. Regardless, perceptions of heat and subsequent exposure to UVR may prove to be an effective supplemental approach to skin cancer prevention. 

## Figures and Tables

**Table 1:** Monthly values of climate variables of the research location.

Climate Variable	Month											
	Jan.	Feb.	Mar.	Apr.	May	Jun.	Jul.	Aug.	Sept.	Oct.	Nov.	Dec.
Average Maximum Temperature (°C)	11.8	13.5	18.3	23.1	27.3	30.7	32.3	31.7	28.3	23.3	17.6	12.8
Average Minimum Temperature (°C)	0.7	1.7	5.6	9.7	14.5	18.7	20.8	20.4	17.1	10.7	5.2	1.8
Average Daily Temperature (°C)	6.3	7.6	11.9	16.4	20.9	24.8	26.6	26.1	22.7	17.0	11.4	7.3
Average Daily Relative Humidity (%)	69	65	64	63	69	72	74	76	75	73	71	70
Average Percentage of Possible Sunshine	49	54	58	66	68	67	63	75	64	63	58	50
Average Number of Days with Clear Skies	8.7	8.6	9.2	10.3	8.7	8.1	6.6	8	9.5	13.3	11.5	9.4
Average Number of Days with Partly Cloudy Skies	6.9	6.3	7.4	8.4	10.5	11.8	12.8	12.5	9.1	7.1	5.9	6

Note: The statistics in this table are based upon meteorological observations taken at Athens, Georgia from 1988 to 2017.

**Table 2:** Mean number of days per nonth and (%) with UV radiation in each category from 1995 to 2017 at Atlanta, Georgia.

UV Index Category	Month												Year (%)
	Jan.	Feb.	Mar.	Apr.	May	Jun.	Jul.	Aug.	Sept.	Oct.	Nov.	Dec.	
*Low* (UVI = 0 to 3)	1.5 (5.2)	0 (0)	0 (0)	0 (0)	0 (0)	0 (0)	0 (0)	0 (0)	0 (0)	0 (0)	0.04 (0.2)	3.3 (11.5)	(1.4)
*Moderate* (UVI = 3 to 5)	29.1 (94.8)	23.4 (85.0)	3.6 (12.0)	0 (0)	0 (0)	0 (0)	0 (0)	0 (0)	0 (0)	13.1 (42.6)	29.6 (99.8)	25.5 (88.5)	(35.1)
*High* (UVI = 6 to 7)	0 (0)	4.1 (14.8)	18.1 (60.0)	3.4 (11.7)	0.2 (0.6)	0 (0)	0 (0)	0 (0)	5.9 (20.6)	17.3 (56.2)	0 (0)	0 (0)	(13.8)
*Very High* (UVI = 8 to 10)	0 (0)	0.04 (0.2)	8.4 (28.0)	24.1 (82.0)	22.1 (72.8)	9.7 (33.2)	7.5 (25.3)	22.5 (76.0)	22.7 (78.9)	0.3 (1.1)	0 (0)	0 (0)	(33.1)
*Extreme* (UVI > 10)	0 (0)	0 (0)	0 (0)	1.8 (6.4)	8.1 (26.6)	19.6 (66.8)	22.2 (74.7)	7.1 (24.0)	0.13 (0.5)	0 (0)	0 (0)	0 (0)	(16.4)

Note: The statistics in this table are based upon clear-sky ultraviolet radiation forecasts for the Atlanta, Georgia UV monitoring station. This data can be found online at: ftp.cpc.ncep.noaa.gov/long/uv/cities.

**Table 3:** Sample characteristics.

Variable	*N*	%
Sex		
Male	430	30.7
Female	970	69.3
Race		
African American	86	6.2
Asian American	114	8.2
Caucasian American	1034	74.0
Hispanic American	49	3.5
Other	115	8.2
Any attempt to suntan this season?		
Yes	685	48.9
No	715	51.1
Reaction of skin when outside for one hour in the middle of the day for the first time in summer without sunscreen		
Burn then peel	388	27.7
Burn then tan	561	40.1
Tan only	451	32.2
To what extend do you like to get a suntan?		
Do not like getting a suntan at all	295	21.1
Like getting a suntan very little	231	16.5
Somewhat like getting a suntan	355	25.4
Like getting a suntan	319	22.8
Very much like getting a suntan	200	14.3
Typical sun exposure behavior when strong summer sunlight is available in a tropical location		
Sun bathe several hours each day	182	13.0
Casual sun exposure only	476	34.0
Use sun protection	670	47.9
Avoid the sun at all times	72	5.1
Level of tan at the end of summer or after a holiday that involves sun exposure		
A dark tan	638	45.6
A medium tan	531	37.9
A light tan	198	14.1
Practically no tan	33	2.4
Ever use a tanning salon?		
Yes	313	22.4
No	1087	77.6
Use of a tanning salon this season		
Yes	96	30.8
No	216	69.2

**Table 4:** Dislike of heat and hot conditions according to typical sun exposure behavior.

Typical Sun Exposure Behavior	*N*	M	SD
Sun bathe several hours each day	182	41.1	14.6
Casual sun exposure only *	476	49.5	14.9
Use sun protection *	670	50.9	15.5
Avoid the sun at all times	72	65.3	9.9

Note: All sun exposure behavior conditions differed significantly (*p* < 0.0001) from each other on the dislike of heat and hot conditions except those marked with *.

## Supplementary Materials

The following are available online at http://www.mdpi.com/1660-4601/15/10/2161/s1, Heat Dislike Scale.

## References

[1] United States Department of Health and Human Services The Surgeon General’s Call to Action to Prevent Skin Cancer. http://www.surgeongeneral.gov.

[2] United States Cancer Statistics Working Group United States Cancer Statistics: 1999–2014 Incidence and Mortality Web-Based Report. https://nccd.cdc.gov/uscs/.

[3] Holman D.M., Freeman M.B., Shoemaker M.L. (2018). Trends in melanoma incidence among non-hispanic whites in the United States, 2005 to 2014. JAMA Dermatol..

[4] Karimkhani C., Green A., Nijsten T., Weinstock M., Dellavalle R., Naghavi M., Fitzmaurice C. (2017). The global burden of melanoma: Results from the Global Burden of Disease Study 2015. Br. J. Derm..

[5] Armstrong B.K., Kricker A. (2001). The epidemiology of UV induced skin cancer. Photochem. Photobiol. B Biol..

[6] Gandini S., Sera F., Cattaruzza M.S., Pasquini P., Picconi O., Boyle P., Melchi C.F. (2005). Meta-analysis of risk factors for cutaneous melanoma: II. Sun exposure. Eur. J. Cancer.

[7] Chang Y., Barrett J.H., Bishop D.T., Armstrong B.K., Bataille V., Bergman W., Berwick M., Bracci P.M., Elwood J.M., Ernstoff M.S. (2009). Sun exposure and melanoma risk at different latitudes: A pooled analysis of 5700 cases and 7216 controls. Int. J. Epidem..

[8] Geller A.C. (2018). The story behind the sharp decline in US tanning bed rates. Am. J. Public Health.

[9] Nadalin V., Marrett L.D., Cawley C., Minaker L.M., Manske S. (2018). Intentional tanning among adolescents in seven Canadian provinces: Provincial comparisons (CRAYS 2015). Prev. Med..

[10] Jin Q., Holman D.M., Jones S.E., Berkowitz Z., Guy G.P. (2018). State indoor tanning laws and prevalence of indoor tanning among US high school students, 2009–2015. Am. J. Public Health.

[11] Kimlin M.G., Lucas R.M., Harrison S.L., van der Mei I., Armstrong B.K., Whiteman D.C., Kricker A., Nowak M., Brodie A.M., Sun J. (2014). The contributions of solar ultraviolet radiation exposure and other determinants to serum 25-hydroxyvitamin D concentrations in Australian adults: The AusD Study. Am. J. Epidemiol..

[12] Downs N.J., Parisi A.V., Igoe D.P. (2014). Measurements of occupational ultraviolet exposure and the implications of timetabled yard duty for school teachers in Queensland, Australia: Preliminary results. Photochem. Photobiol. B Biol..

[13] Sun J., Lucas R.M., Harrison S., van der Mei I., Armgstrong B.K., Nowak M., Brodie A., Kimlin M.G. (2014). The relationship between ambient ultraviolet radiation (UVR) and objectively measured personal UVR exposure dose is modified by season and latitude. Photochem. Photobiol. Sci..

[14] Parsons K. (2014). Human Thermal Environments.

[15] Morabito M., Grifoni D., Crisci A., Fibbi L., Orlandini S., Gensini G.F., Zipoli G. (2014). Might outdoor heat stress be considered a proxy for the unperceivable effect of the ultraviolet-induced risk of erythema in Florence?. J. Photochem. Photobiol. B Biol..

[16] Fiala D., Havenith G., Bröde P., Kampmann B., Jendritzky G. (2012). UTCI-Fiala multi-node model of human heat transfer and temperature regulation. Int. J. Biometeorol..

[17] Höppe P. (1997). Aspects of human biometeorology in past, present, and future. Int. J. Biometeorol..

[18] Auliciems A. (1981). Towards a psycho-physiological model of thermal perception. Int. J. Biometeorol..

[19] de Dear R.J., Brager G.S. (1998). Developing an adaptive model of thermal comfort and preference. ASHRAE Trans..

[20] Knez I., Thorsson S. (2006). Influences of culture and environmental attitude on thermal emotional and perceptual evaluations of a public square. Int. J. Biometeorol..

[21] Yang W., Wong N.H., Jusuf S.K. (2013). Thermal comfort in outdoor urban spaces in Singapore. Build. Environ..

[22] Malchaire J., Piette A., Kampmann B., Mehnert P., Gebhardt H., Havenith G., den Hartog E., Holmer I., Parsons K., Alfano G. (2001). Development and validation of the predicted heat strain model. Ann. Occup. Hyg..

[23] Johnstone A.M., Murison S.D., Duncan J.S., Rance K.A., Speakman J.R. (2005). Factors influencing variation in basal metabolic rate include fat-free mass, fat mass, age, and circulating thyroxine but not sex, circulating leptin, or triiodothyronine. Am. J. Clin. Nutr..

[24] Parsons K.C., Hamley E.L., Mercer J.B. (1985). Practical methods for the estimation of human metabolic heat production. Thermal Physiology.

[25] Knez I., Thorsson S., Eliasson I., Lindberg F. (2009). Psychological mechanisms in outdoor place and weather assessment: Towards a conceptual model. Int. J. Biometeorol..

[26] Stewart A.E. (2009). Minding the weather: The measurement of weather salience. Bull. Am. Meteor. Soc..

[27] Kottek M., Grieser J., Beck C., Rudolf B., Rubel F. (2006). Map of the Köppen-Geiger climate classification updated. Meteor Zeit..

[28] National Center for Environmental Information 2017 Local Climatological Data: Annual Summaries with Comparative Data—Athens, Georgia. https://www.ncdc.noaa.gov/IPS/lcd/lcd.html.

[29] World Health Organization, World Meteorological Organization, United Nations Environment Programme, International Commission on Non-Ionizing Radiation Protection (2002). Global Solar UV Index: A Practical Guide.

[30] Climate Prediction Center, National Center for Environmental Prediction UV Index: Annual Time Series. http://www.cpc.ncep.noaa.gov/products/stratosphere/uv_index/uv_annual.shtml.

[31] UV-B Monitoring and Research Program United States Department of Agriculture/Colorado State University, Fort Collins, Colorado. http://uvb.nrel.colostate.edu/UVB/index.jsf.

[32] Maio G., Haddock G. (2015). The Psychology of Attitudes and Attitude Change.

[33] Gershenwald J.E., Guy G.P. (2016). Stemming the rising incidence of melanoma: Calling prevention to action. J. Natl. Cancer Inst..

[34] Chang C., Murzaku E.C., Penn L., Abbasi N.R., Davis P.D., Berwick M., Polsky D. (2014). More skin, more sun, more tan, more melanoma. Am. J. Public Health.

