# Supplementary material for: The Dislike of Hot Thermal Conditions and Its Relationship with Sun (Ultraviolet Radiation) Exposure in the Southeastern United States
Source: Int J Environ Res Public Health. 2018 Oct 1;15(10):2161. doi: 10.3390/ijerph15102161 (PMC6210274; doi:10.3390/ijerph15102161)
Supplement: Supplementary file 1 [file ijerph-15-02161-s001.pdf]

Heat Dislike Scale

© 2017 by Alan E. Stewart and Michael G. Kimlin

Instructions: The statements below are about the sensations and perceptions people may have of warm weather while outside. Please read each statement and then indicate the extent to which you disagree or agree with each one based upon how you tend to feel when outside during warm weather. For the purposes of responding to these items, *warm* or *hot* pertain to how you define these conditions for yourself.

Please using the following scale for your responses and click the button of the alternative the represents your choice.

Strongly disagree
Somewhat disagree
Neither agree nor disagree
Somewhat agree
Strongly agree

1. Being outside in hot weather is unpleasant for me.
2. I avoid being in out in the sun during warm weather because the sunlight makes me feel too hot.
3. I do not like the way that hot air temperatures make me feel.
4. Being outside in hot weather does not bother me.*
5. I avoid being outside during hot weather spells because I do not like the sensation of being hot.
6. The sensation of becoming hot is unpleasant to me.
7. I prefer being outside when the weather is cool rather than warm.
8. I do not go outside in the summer as much as other people do because I get hot easily.
9. I do not like the sensation of feeling hot.
10. I only like being outside in the sun when the air temperature is cool.
11. The sensation of feeling hot does not bother me.*
12. Being outside in warm weather makes me feel uncomfortably hot.
13. I tend to do less outside when the weather is warm because getting hot is a hassle.
14. It is more comfortable for me to be outside when the sky is cloudy than when it is sunny.
15. I enjoy being outside in the sun and feeling hot.*

<u>Scoring</u>:
The rating scale alternatives have the following values for scoring purposes:

Strongly disagree=1
Somewhat disagree=2
Neither agree nor disagree=3
Somewhat agree=4
Strongly agree=5

*Note that items 4, 11, and 15 are reverse-scored.

The total Heat Dislike score is the sum of the item responses. Higher scores correspond to a greater dislike of warm/hot conditions.
